# Adherence to Systemic Therapy for Atopic Dermatitis in Adult Patients in Saudi Arabia: A Cross-Sectional Study

**DOI:** 10.7759/cureus.72866

**Published:** 2024-11-02

**Authors:** Ali M Aleid, Raghad A Alharbi, Nafisah M Al Radhwan, Nawaf S Alsulamy, Maryam A Alzahrani, Retaj W Calacattawi, Norah H Alsultan, Awatif M Alrasheeday, Faiza Aljarameez, Abbas Al Mutair

**Affiliations:** 1 Dermatology, Ministry of Health, Riyadh, SAU; 2 Medicine, Almaarefa University, Diriyah, SAU; 3 Internal Medicine, Medical University of Warsaw, Warsaw, POL; 4 Medicine, King Abdulaziz University, Jeddah, SAU; 5 Medicine, University of Hail, Hail, SAU; 6 Medicine, King Saud bin Abdulaziz University for Health Sciences, Riyadh, SAU; 7 Medicine, King Faisal University, Hofuf, SAU; 8 Nursing, University of Hail, Hail, SAU; 9 Critical Care Medicine, King Saud bin Abdulaziz University for Health Sciences, King Abdullah International Medical Research Center, Ministry of the National Guard Health Affairs, Riyadh, SAU; 10 Research Center, Almoosa Specialist Hospital, Al Mubarraz, SAU; 11 Nursing, University of Wollongong, Wollongong, AUS; 12 Nursing, Almoosa College of Health Sciences, Al Mubarraz, SAU; 13 Nursing, Prince Sultan Military College for Health Sciences, Dammam, SAU; 14 Medical-Surgical Nursing, Princess Nourah bint Abdulrahman University, Riyadh, SAU

**Keywords:** atopic dermatitis, disease severity, healthcare provider monitoring, medication adherence, quality of life, systemic pharmacological interventions

## Abstract

Introduction: Atopic dermatitis (AD) is a chronic inflammatory skin disease that significantly impacts both pediatrics and adults. Despite the availability and proven efficacy of systemic medications, there remains a critical issue of non-adherence among patients. This study aimed to assess adherence to systemic therapy among Saudi adult patients with AD.

Methods: The study employed a cross-sectional study with an online questionnaire that was distributed among a convenience sample of 1,154 adult patients with AD in Saudi Arabia. In addition to descriptive statistical analysis, Pearson's correlation and chi-squared tests were used to determine the correlations between adherence to systemic therapy for AD and its predictable factors.

Results: AD was diagnosed over five years in 61.3% of the participants. The vast majority of those diagnosed with AD (73%) were prescribed systemic medications, with a moderate level of adherence to this therapy. While adherence to systemic therapy significantly correlated with improved quality of life, factors including forgetfulness, side effect concerns, patient’s belief in medication importance, disease severity, and medication complexity impacted adherence to this mode of therapy. Factors including patient’s belief in medication importance, disease severity, and medication complexity impacted adherence.

Conclusion: The findings of this study highlight the need for multifaceted interventions to improve adherence to systemic therapy for AD. Addressing patient concerns about the use of systemic therapy for AD and reinforcing the importance of medication use for long-term disease management and quality-of-life improvement is recommended to explore the barriers to adherence and enhance compliance to systemic therapy in this population.

## Introduction

Atopic dermatitis (AD), also known as eczema, is a chronic inflammatory skin disease that significantly impacts both children and adults. The exact pathological mechanism is unknown, but it is believed to be a result of an interaction of genetic and environmental factors. The pathological interactions of a defective skin barrier, immune system abnormalities, genetic predisposition, and environmental exposure to allergens lead to a cycle of local and systemic inflammatory reactions. The most prominent symptom of AD is intense itchiness and is characterized by several key features including erythematous patches and dry cracked skin. Intense scratching can lead to skin irritation known as excoriation and chronic scratching can result in thickened, leathery skin, known as lichenification.

AD is often managed through a combination of trigger avoidance and topical treatments such as steroids and emollients. Alternative therapies, such as phototherapy, can be an effective option when topical medications alone are insufficient, particularly for individuals with skin of color [1]. In cases of moderate to severe AD, both biologic and non-biologic systematic treatments become essential [2]. The most common systemic medications approved for the treatment of AD include oral corticosteroids (such as prednisone), immunomodulators (such as pimecrolimus, tacrolimus, and cyclosporine), and biologics targeting certain monoclonal antibodies involved in the inflammation cycle (such as Dupixent (dupilumab) and Trinarize (trinemalizumab). Nevertheless, non-adherence to these treatments remains a pervasive problem that leads to poor outcomes and symptom exacerbation. Medication non-adherence is a factor directly linked to resistance and poor therapeutic outcomes [3,4]. This non-compliance not only adds to the suffering of patients but also puts a strain on healthcare systems and frequently results in unwarranted escalation in medication intake [5]. Influential factors on this compliance issue may include health literacy, patient-provider communication, medication cost, and access to healthcare facilities [6].

Historically, AD was first described in 1933 by Fred Wise (1881-1950) and Marion Sulzberger (1895-1983), marking the beginning of clinical recognition of the term AD in dermatology. Since then, numerous studies have investigated its impact and prevalence worldwide. Globally, the condition affects approximately 25% of children and up to 10% of adults [7]. In Saudi Arabia, recent studies have revealed a prevalence rate of 6% to 12%, highlighting the ongoing significance and widespread nature of AD in various populations [8]. The rising prevalence of AD, especially among young adults in Saudi Arabia, underscores the importance of early diagnosis and effective treatment in preventing disease spread and improving life quality [8-11]. Accordingly, this study hypothesized a low level of compliance to systemic treatment among AD patients in Saudi Arabia, attributable to challenges such as ineffective communication, inadequate patient education, and limited availability or accessibility of therapeutic agents. Moreover, it is anticipated that there is a significant correlation between adherence levels and treatment outcomes, aligning with global trends [12]. Thus, this study primarily aimed to assess the adherence level to systemic medications for AD among adult patients in Saudi Arabia. In addition, the study aimed to determine the factors influencing adherence and to assess the correlation between adherence to systemic medications and treatment outcomes, including symptom control and quality of life. This research study serves as a vital call to action, spotlighting the significant health implications of AD and the necessity of consistent treatment adherence. Additionally, the study is crucial in highlighting the challenges affecting adherence to systemic treatment among AD patients in Saudi Arabia, emphasizing the disease's prevalence and the need for collective efforts by healthcare institutions, the government, and the public to improve adherence to treatment protocols.

## Materials and methods

Study design

The study employed a cross-sectional design to examine patterns of adherence to systemic medication among adult patients with AD in Saudi Arabia.

Study participants

Participants were adults (18 years and older) with a medical diagnosis of AD and receiving systemic medications for the management of their condition. The ability to comprehend and answer the study survey was one of the eligibility requirements, along with a verified diagnosis of AD and a prescription for systemic medication for at least six months. Patients who have been taking systemic medications for at least six months are more likely to have established patterns of adherence and experience the long-term effects of their treatment. This can provide more valuable insights into the factors influencing medication adherence, the long-term impact of adherence on disease management, and potential strategies for improving adherence rates. To ensure that the study focused exclusively on AD patients undergoing systemic pharmacological interventions and minimized potential confounding factors, individuals with conditions or factors that could significantly impact their adherence to systemic therapy, such as severe cognitive impairments, were excluded from the study.

The required sample size was computed based on a 50% population proportion expected for adherence levels, a 5% margin of error, and a 95% confidence level. A sample size of 1,000 was determined. The target sample was raised to 1,200 with the assumption of a 20% non-response rate. The online survey was completed by 1,154 participants, which provided a sufficient sample size for the research. To create a representative sample, cluster random sampling was employed to select roughly equal numbers of participants from each geographical region.

Participants were recruited from dermatology and asthma-allergy clinics in Saudi Arabia. This study was approved by the Institutional Review Board and Research Ethics Committee of King Faisal University in Hofuf, Saudi Arabia, with reference number 1393. After receiving institutional ethical approval, a self-administered electronic validated questionnaire was distributed to gather participants' information.

Study variables

The questionnaire gathered data on disease characteristics, medication usage patterns, adherence barriers, quality of life indicators, and beliefs regarding the significance of medications, in addition to demographic information. Adherence to systemic medication regimens was the primary outcome variable. Based on the patient's self-reported frequency of medication intake, adherence was evaluated in accordance with prescription guidelines. The predictor variables included demographic factors such as age, gender, education level, employment status, income, and geographic location. Disease-related factors were also assessed, including the duration of the disease, intensity of symptoms, and history of hospitalization. Medication-related factors encompassed the quantity of medications prescribed, experienced adverse effects, comprehension of medication directions, and experiences with medication refills. Additionally, patients' perceptions of the importance of their medications were assessed, along with adherence-related obstacles and promoters. The quality and frequency of communication between patients and healthcare providers were also evaluated. Covariates for quality of life included metrics such as the level of symptom control and contentment regarding general well-being. Further variables included gains perceived since beginning medication, the frequency of flare-ups, and necessary lifestyle changes for health management. The levels of adherence were categorized as excellent (>90% of doses taken), subpar (75-90%), and low (<75%). Where a 5-point Likert scale was used to rate the patient’s beliefs, the frequency and intensity of symptoms were used to rate the severity of the disease as mild, moderate, or severe. The severity of AD was assessed using the scoring atopic dermatitis (SCORAD) index. The SCORAD system is a commonly used severity rating scale that measures the extent, intensity, and subjective symptoms (such as itching and sleeplessness) of the disease. The SCORAD index is calculated based on the following criteria: (1) extent, measured by using the "rule of nines" to determine the affected skin surface area; (2) intensity, assessed by rating symptoms like redness, swelling, oozing/crusting, excoriation, lichenification, and dryness; and (3) subjective symptoms, including patient-reported levels of itching and sleeplessness.

Statistical analysis

The data were analyzed using SPSS Statistics version 29 (IBM Corp. Released 2023. IBM SPSS Statistics for Windows, Version 29.0.2.0 Armonk, NY: IBM Corp.). Descriptive analyses were used to obtain frequencies and percentages of respondents’ characteristics. Multi-item scales, such as symptom control and understanding levels, were summarized using mean scores and standard deviations. Pearson's correlation and chi-squared tests were used to determine the correlation between medication factors, severity, beliefs, quality of life, and therapy adherence.

## Results

Demographic characteristics of participants

The demographic characteristics of the study participants are presented in Table 1. The sample has a near-equal split between females (51.3%) and males (48.7%) with almost even distribution across age groups, with the highest representation in the 35-44 (29.3%) and 45-54 (28.2%) age ranges. The majority of the sample hold a bachelor’s degree (41.1%), followed by those participants with a high school diploma or less (31.9%). Most of the participants are unemployed (36.0%), with (31.7%) employed full-time, and almost half of the participants (47.3%) reported a monthly income of less than SAR 3,000. A significant portion of the participants reside in urban areas (71.1%), followed by suburban (18.7%) and rural areas (10.2%).

**Table 1:** Sample demographic characteristics SAR: Saudi Riyal

		Count	%
Age (years)	18-24	152	14.12%
	25-34	178	15.4%
	35-44	338	29.3%
	45-54	326	28.2%
	Above 65	160	13.9%
Gender	Female	592	51.3%
	Male	562	48.7%
Education level	Haigh school or less	368	31.9%
	Diploma	216	18.7%
	Bachelor's degree	474	41.1%
	Doctorate or higher	56	4.9%
	Master's degree	40	3.5%
Employment status	Student	92	8.0%
	Unemployed	416	36.0%
	Retired	120	10.4%
	Employed part-time	160	13.9%
	Employed full-time	366	31.7%
What is your monthly income? (SAR)	SAR 3,000-6,000	340	29.5%
	SAR 6,000-9,000	100	8.7%
	Less than SAR 3,000	546	47.3%
	More than SAR 9,000	168	14.6%
Residence area	South province	120	10.4%
	Eastern province	354	30.7%
	Northern province	240	20.8%
	Western province	240	20.8%
	Middle province	200	17.3%
Geographic location	Urban	820	71.1%
	Rural	118	10.2%
	Suburban	216	18.7%

Clinical information on systemic medications in the treatment of atopic dermatitis

Table 2 provides the results of participants' experiences with AD and their use of systemic medications. Most of the participants (80.3%) have been diagnosed with AD for more than five years, with a significant portion (42.5%) having it for one to five years. Almost half of the participants (56.0%) rated AD severity as being “moderate,” followed by mild (34.0%) and severe (10.1%), with the majority (73.3%) having been prescribed systemic medications for their condition. Participants reported an average moderate understanding of the importance of medication adherence (M=5.5, SD=3.027), indicating significant variation among participants. A significant portion reported sometimes forgetting to take medications (54.8%) and expressed concerns about side effects (32.2%), potentially influencing adherence. Satisfaction with medication effectiveness varied, with the majority (46.1%) being moderately satisfied. Forgetfulness (51.6%) is the most reported barrier to medication adherence, followed by cost (14.2%) and complex medication regimens (13.7%). Fear of side effects (11.1%) and lack of information (3.6%) are also other reported challenges to medication adherence. The majority (66.6%) perceived the communication about their medications as fair, whereas a small portion (1.7%) perceived it as excellent. The majority (69.2%) believed that adherence is crucial for managing AD, suggesting a positive attitude towards treatment.

**Table 2:** Clinical information on systemic medications in the treatment of atopic dermatitis SD: standard deviation

		Count	%
How long have you been diagnosed with atopic dermatitis?	1-5 years	490	42.5%
	5-10 years	436	37.8%
	More than 10 years	228	19.8%
How would you rate the severity of your atopic dermatitis?	Mild	392	34.0%
	Severe	116	10.1%
	Moderate	646	56.0%
Have you ever been prescribed systemic medications (e.g., oral immunosuppressants or biologics) for your atopic dermatitis?	No	308	26.7%
	Yes	846	73.3%
On a scale of 1-10, how well do you understand the importance of adherence to systemic medications for treating atopic dermatitis? (mean ± SD)		5.5 ± 3.027	
How often do you forget to take your systemic medications as prescribed?	Never	36	3.1%
	Sometimes	632	54.8%
	Always	40	3.5%
	often	198	17.2%
	Rarely	248	21.5%
Are you concerned about the potential side effects of your systemic medications?	No	646	56.0%
	Not sure	136	11.8%
	Yes	372	32.2%
How satisfied are you with the effectiveness of your current systemic medications in managing your atopic dermatitis symptoms?	Moderately satisfied	532	46.1%
	Very satisfied	98	8.5%
	Moderately dissatisfied	40	3.5%
	Neither satisfied nor dissatisfied	484	41.9%
Have you experienced any barriers that make it difficult for you to adhere to your systemic medications?	Fear of side effects	126	11.1%
	Forgetfulness	596	51.6%
	Lack of information about the medications	135	3.6%
	Cost of medications	176	14.2%
	Complex medication regimen	160	13.7%
How would you rate the quality of communication and support provided by your healthcare provider regarding your systemic medications?	Good	226	19.6%
	poor	140	12.1%
	Fair	768	66.6%
	Excellent	20	1.7%
Do you believe that adhering to your systemic medications is crucial for effectively managing your atopic dermatitis?	No	300	26.0%
	Not sure	56	4.9%
	Yes	798	69.2%

Adherence to systemic therapy and quality of life

Table 3 delves into participants’ experiences with symptom control, quality of life, and overall management of AD while using systemic therapy. Participants rated their symptom control with current systemic medications with an average mean score of 5.78 with a significant variation in individual experience (SD=3.073). When asked about their satisfaction with the overall quality of life while adhering to systemic medications, 47.5% (N=548) reported being moderately satisfied, while 18.5% (N=214) were very satisfied. Regarding symptom improvement since starting systemic medications, 64.3% (N=742) experienced minor improvements, and 35.7% (N=412) reported significant improvements. Despite therapy adherence, a significant portion of the participants (64.6%, N=746) reported experiencing occasional flare-ups, and a significant majority of the sample (77.3%, N=892) have had to make lifestyle modifications due to AD even while adhering to medications.

**Table 3:** Adherence to systematic therapy and quality of life of patients with atopic dermatitis SD: standard deviation

		Count	%
On a scale of 1-10, rate the level of symptom control you experience with your current systemic medications. (mean ± SD)		5.78 ± 3.073	
How satisfied are you with your overall quality of life while adhering to your systemic medications?	Moderately satisfied	548	47.5%
	Very satisfied	214	18.5%
	Moderately dissatisfied	100	8.7%
	Neither satisfied nor dissatisfied	292	25.3%
Have you noticed any improvements in your symptoms since starting your systemic medications?	Yes, minor improvements	742	64.3%
	Yes, significant improvements	412	35.7%
How frequently do you experience flare-ups of your atopic dermatitis despite adhering to your systemic medications?	Frequently	94	8.1%
	Occasionally	746	64.6%
	Rarely	314	27.2%
Have you had to make any lifestyle modifications due to your atopic dermatitis while adhering to your systemic medications?	No	262	22.7%
	Yes	892	77.3%

Correlation between adherence to systemic therapy and quality of life

The study results indicate a strong positive correlation (r=0.753, p<0.001) between adherence to systemic therapy and quality of life. This suggests that better adherence to systemic medications is associated with higher quality of life among patients with AD.

Disease severity and adherence to systemic medications

Table 4 presents the frequency and severity of AD symptoms, as well as the impact on patients' lives. While a significant portion of the sample (17.0%) experience frequent symptoms (6-10 times per month), and 5.0% experience symptoms almost daily, the vast majority of the participants (57.9%) experience AD symptoms occasionally (three to five times per month). Most of the patients (48.4%) have been hospitalized due to severe AD symptoms, highlighting the impact of the condition on daily life. Itchiness is a prevalent symptom, with 32.2% experiencing it frequently and 52.9% occasionally. Because of the severity of their symptoms, the majority (79.0%) have needed higher doses of systemic medications, indicating the need for dose adjustments to effectively manage the condition.

**Table 4:** Disease severity and adherence to systemic medications in patients with atopic dermatitis

		Count	%
How frequently do you experience symptoms of atopic dermatitis on a monthly basis?	Frequently (6-10 times)	196	17.0%
	Always (more than 10 times)	58	5.0%
	Occasionally (3-5 times)	668	57.9%
	Rarely (1-2 times)	232	20.1%
Have you ever been hospitalized due to severe atopic dermatitis symptoms?	No	596	51.6%
	Yes	558	48.4%
How often do you experience itchiness associated with your atopic dermatitis?	Frequently	372	32.2%
	Always	36	3.1%
	Occasionally	610	52.9%
	Rarely	136	11.8%
Have you ever been prescribed higher doses of systemic medications due to the severity of your atopic dermatitis symptoms?	No	242	21.0%
	Yes	912	79.0%

Association between adherence and disease severity

Table 5 examines the relationship between the belief in the importance of adhering to systemic medications and various factors related to AD. The vast majority (69.2%) believe that adhering to systemic medications is crucial for managing AD. Patients who experience more frequent symptoms (frequently or always) are significantly more likely to believe in the importance of medication adherence compared to those with occasional or rare symptoms. Similarly, those who have been hospitalized due to severe AD symptoms or those who experience frequent or always itchiness (67.7%, N=252) or required higher medication doses due to severe symptoms (85.1%, N=206) are significantly more likely to believe in the importance of medication adherence.

**Table 5:** Association between therapy adherence and disease severity in patients with atopic dermatitis

		Do you believe that adhering to your systemic medications is crucial for effectively managing your atopic dermatitis?						p-value
		No		Not sure		Yes		
		Count	Row N%	Count	Row N%	Count	Row N%	
How frequently do you experience symptoms of atopic dermatitis on a monthly basis?	Frequently (6-10 times)	60	30.6%	0	0.0%	136	69.4%	<0.001
	Always (more than 10 times)	20	34.5%	0	0.0%	38	65.5%	
	Occasionally (3-5 times)	180	26.9%	56	8.4%	432	64.7%	
	Rarely (1-2 times)	40	17.2%	0	0.0%	192	82.8%	
Have you ever been hospitalized due to severe atopic dermatitis symptoms?	No	100	16.8%	56	9.4%	440	73.8%	<0.001
	Yes	200	35.8%	0	0.0%	358	64.2%	
How often do you experience itchiness associated with your atopic dermatitis?	Frequently	100	26.9%	20	5.4%	252	67.7%	<0.001
	Always	0	0.0%	0	0.0%	36	100.0%	
	Occasionally	200	32.8%	36	5.9%	374	61.3%	
	Rarely	0	0.0%	0	0.0%	136	100.0%	
Have you ever been prescribed higher doses of systemic medications due to the severity of your atopic dermatitis symptoms?	No	0	0.0%	36	14.9%	206	85.1%	<0.001
	Yes	300	32.9%	20	2.2%	592	64.9%	

Medication-related factors and adherence to systemic medications

Table 6 examines medication-related factors and their correlation with adherence to systemic medications among patients with AD. The majority of patients (52.2%) are taking only one systemic medication for their condition, while 42.8% are taking two, and a smaller percentage (5.0%) are on three or more medications. The frequency of experiencing side effects varied, with the majority (44.5%, N=514) experiencing side effects occasionally. While a majority (52.5%) have a moderate understanding of medication instructions, a combined 17.2% either have no understanding or only a limited understanding. For medication adherence, a significant number of patients (39.2%) run out of medication before refills at least sometimes, and another significant proportion (78.3%) have stopped taking medications without consulting their healthcare provider.

**Table 6:** Association between therapy adherence and disease severity in patient with atopic dermatitis

		Do you believe that adhering to your systemic medications is crucial for effectively managing your atopic dermatitis?						p-value
		No		Not sure		Yes		
		Count	Row N %	Count	Row N %	Count	Row N %	
How frequently do you experience symptoms of atopic dermatitis on a monthly basis?	Frequently (6-10 times)	60	30.6%	0	0.0%	136	69.4%	<0.001
	Always (more than 10 times)	20	34.5%	0	0.0%	38	65.5%	
	Occasionally (3-5 times)	180	26.9%	56	8.4%	432	64.7%	
	Rarely (1-2 times)	40	17.2%	0	0.0%	192	82.8%	
Have you ever been hospitalized due to severe atopic dermatitis symptoms?	No	100	16.8%	56	9.4%	440	73.8%	<0.001
	Yes	200	35.8%	0	0.0%	358	64.2%	
How often do you experience itchiness associated with your atopic dermatitis?	Frequently	100	26.9%	20	5.4%	252	67.7%	<0.001
	Always	0	0.0%	0	0.0%	36	100.0%	
	Occasionally	200	32.8%	36	5.9%	374	61.3%	
	Rarely	0	0.0%	0	0.0%	136	100.0%	
Have you ever been prescribed higher doses of systemic medications due to the severity of your atopic dermatitis symptoms?	No	0	0.0%	36	14.9%	206	85.1%	<0.001
	Yes	300	32.9%	20	2.2%	592	64.9%	

Association between medication-related factors and adherence to systemic medications

Table 7 explores the relationship between the belief in the importance of adhering to systemic medications for AD management and various factors related to medication use, side effects, and treatment experience. A significant majority (69.2%) of patients believe that adhering to systemic medications is crucial for effectively managing AD. Patients using more than one systemic medication (two or more) are significantly more likely to believe in the importance of adherence compared to those using only one medication. Patients who frequently experience side effects (69.0%) are somehow less likely to believe in the importance of medication adherence as compared to those who rarely experience side effects (79.0%), who reported more likelihood to believe in adherence (p<0.001). Those who have a good understanding of their medication instructions (moderately well or very well) and who never or rarely run out of medication, as well as those who have never stopped taking their medication without consulting a healthcare provider, are significantly more likely to believe in the importance of adherence (p<0.001).

**Table 7:** Association between medication-related factors and adherence to systemic medications in patients with atopic dermatitis

		Do you believe that adhering to your systemic medications is crucial for effectively managing your atopic dermatitis?						p-value
		No		Not sure		Yes		
		Count	N %	Count	N %	Count	N %	
How many different systemic medications do you take for your atopic dermatitis?	Two	120	24.3%	20	4.0%	354	71.7%	0.088
	Three or more	20	34.5%	0	0.0%	38	65.5%	
	One	160	26.6%	36	6.0%	406	67.4%	
How frequently do you experience side effects from your systemic medications?	Frequently	60	23.3%	20	7.8%	178	69.0%	<0.001
	Always	20	100.0%	0	0.0%	0	0.0%	
	Occasionally	180	35.0%	0	0.0%	334	65.0%	
	Rarely	40	11.0%	36	9.9%	286	79.0%	
How well do you understand the instructions for taking your systemic medications?	Moderately well	160	26.4%	18	3.0%	428	70.6%	<0.001
	Very well	80	22.9%	18	5.1%	252	72.0%	
	Not at all	40	40.0%	20	20.0%	40	40.0%	
	Not very well	20	20.4%	0	0.0%	78	79.6%	
How often do you run out of your systemic medications before obtaining a refill?	Never	80	40.4%	0	0.0%	118	59.6%	<0.001
	Always	0	0.0%	38	100.0%	0	0.0%	
	Often	20	33.3%	0	0.0%	40	66.7%	
	Sometimes	120	33.9%	0	0.0%	234	66.1%	
	Rarely	80	15.9%	18	3.6%	406	80.6%	
Have you ever stopped taking your systemic medications without consulting your healthcare provider?	No	40	16.0%	0	0.0%	210	84.0%	<0.001
	Yes	260	28.8%	56	6.2%	588	65.0%	

## Discussion

This research study provides insights into the adherence of adults with AD to systemic medications. Similar to previous research by Churnosov et al., Dvornyk et al., and Elezbawy et al., a positive correlation between therapy adherence and quality of life was found in the present study [13-15]. These findings highlight the importance of adherence in effectively managing symptoms and improving overall well-being in patients with AD. However, the moderate average symptom control rating in the present study, combined with dissatisfaction with the quality of life for a significant portion of participants, suggests room for improvement in treatment effectiveness and management approaches. The prevalence of flare-ups and the need for lifestyle modifications reported by the participants in this study further highlight this point. This is a well-documented point by the findings of this study indicating that AD significantly impacts the lives of patients, with frequent or more severe symptoms, hospitalizations, and the need for medication dose adjustments being common. These findings emphasize that AD significantly impacts the lives of patients, indicating a need for further research to either optimize current therapy or develop more effective treatment strategies to manage AD patients and improve quality of life.

The findings of this study show that while systemic medications are widely used for the treatment of AD, various factors were reported that seem to be affecting medication adherence. Similar to a study by Elezbawy et al., the data in this study suggests a strong statistical correlation between the severity of AD symptoms and the belief in the importance of medication adherence [15]. Patients who experience more severe or frequent symptoms, such as frequent or persistent itchiness and hospitalizations, are more likely to recognize the crucial role of medication in managing their condition. Although a patient’s belief may be necessary, in extreme or unstable cases, beliefs might not be sufficient to ensure adherence, emphasizing the need for specialized support [15]. This implies room for improvement in patient education and communication with healthcare providers regarding medication effectiveness and management approaches. Thus, in order to improve motivation and overcome challenges to adherence, patients with severe symptoms who necessitate higher medication dosages or hospitalization are vulnerable subgroups that should receive intensive education and follow-up [16,17]. This additionally suggests a need for interventions by healthcare providers to further investigate these challenges and address others to improve patient understanding, adherence, and experience with side effects, and ultimately enhance treatment outcomes for patients with AD [18,19].

Furthermore, the findings of the study highlighted several other avoidable obstacles that affect adherence to systemic medications in patients with AD. Comparable to the findings by Jacob et al. and Kharwade et al., the participants in this study reported non-adherence factors such as forgetfulness, misinterpreting instructions, neglecting to get refills, concerns about side effects, and quitting drugs without supervision [20,21]. Specific interventions aiming at resolving these common roadblocks may significantly improve adherence [17]. Over half of the participants identified forgetting as a significant barrier, indicating that this group might benefit from reminder tools [15,17]. Approximately one-third needed to understand the instructions clearly, indicating the need for better education delivery strategies considering each patient’s unique health literacy level. Thus, patient counseling by the treating dermatologist should be thoroughly done before initiating the treatment. Additionally, similar to the findings by Siddiqui, Silverberg et al., and Singh et al., there have been conflicting reports about the providers' communication and assistance with prescription drugs [22-24]. In this study, only about one-fifth of respondents rated such guidance as good or excellent, while two-thirds thought it was fair. Concerns reported in this study were about medication side effects, running out of medicine, or stopping it abruptly without consulting the healthcare provider, which are other examples of how poor communication can compromise continuity of care. To improve outcomes, healthcare providers need to reevaluate their treatment strategies to optimize management approaches and select the proper medications, weighing their benefits and risks [25-27]. This suggests that relationship-centered care to improve patient-healthcare provider rapport probably improves adherence. Appointment scheduling might enable longer durations of medication counseling sessions that facilitate in-depth question-answering [28-30]. Additionally, streamlining dosages, spacing out doses optimally, and minimizing pill burden, if practical, to simplify complex regimens as much as possible might be helpful.

The limitations of this research study include its cross-sectional design, which hinders concluding a causal relationship, as well as the self-reported data, which could be skewed by participants’ responses and recall biases. Provider viewpoints and objective adherence metrics, such as medication possession ratio computations, are suggested to be included in future research. More robustness could be added by longitudinal evaluations tracking changes in outcomes after focused interventions.

## Conclusions

This study reveals the importance of medication adherence in improving symptom control and quality of life for adult patients with atopic dermatitis. Addressing medication non-adherence can potentially improve patient compliance and quality of life, which ultimately reduces the overall burden of atopic dermatitis on healthcare systems and society. In this population, increasing adherence through such strategies focusing on streamlining complicated regimens and improving patient-provider communication has the potential to improve the quality of life and outcomes related to disease control. Future research should focus on developing interventions to enhance adherence and evaluating barriers to adherence and their impact on long-term patient outcomes and healthcare costs.
